# Transdermal metformin hydrochloride-loaded cubic phases: *in silico* formulation optimization, preparation, properties, and application for local treatment of melanoma

**DOI:** 10.1080/10717544.2019.1587046

**Published:** 2019-03-24

**Authors:** Xiang Yu, Wei Zhou, Hongmei Wang, Sheng Lu, Yiguang Jin, Junhui Fu

**Affiliations:** a Department of Pharmacy, First Hospital of Huzhou, First Affiliated Hospital of Huzhou University, Huzhou, China;; b Department of Pharmaceutical Sciences, Beijing Institute of Radiation Medicine, Beijing, China

**Keywords:** Apoptosis, cubic phase, *in silico*, metformin hydrochloride, melanoma, transdermal delivery

## Abstract

Metformin hydrochloride (Met) is commonly used for antidiabetic therapy though its antimelanoma action is also reported. Conventional oral administration method of Met is not appropriate for therapy of melanoma because of large dose, adverse reactions, and low efficiency. Here, a transdermal Met-loaded cubic phase was developed for local treatment of melanoma. *In silico* formulation optimization of the cubic phases was done, and the corresponding formulations were prepared and characterized. The optimized formulations were screened based on the stable microstructure and proper fluidity. Highly efficient mouse skin permeability of Met was found with the cubic phases compared to Met solutions. High antimelanoma effect of transdermal Met-loaded cubic phases also was shown by the significant decrease of tumor volume and the improvement of melanoma cell apoptosis on the B16 melanoma mice. Met-loaded cubic phases are a promising topically applied medication for local therapies of melanoma.

## Introduction

Malignant melanoma is one of the most aggressive forms of skin cancers, which is characterized by early metastasis, rapid development, poor prognosis, and high mortality (Yu et al., 2015). According to the report of the National Cancer Institute, more than 65,000 new cases of melanoma were reported in the United States each year with estimated 8650 deaths (Ferlay et al., 2018). Despite extensive research and partial successes are gained by the use of decarbonize (Mazzio & Soliman, 2018), ipilimumab (Afzal et al., 2018) and interleukin-2 (Ramanujam et al., 2015), currently there is still lack of effective chemotherapy methods for the treatment of invasive melanoma.

Metformin hydrochloride (Met) is a drug commonly used for the treatment of type-2 diabetes. Moreover, numerous studies have demonstrated that Met exhibits anti-melanoma activity. The anti-melanoma effect of Met is mediated through p21- and AMPK-independent cell cycle arrest (Neto et al., 2017), apoptosis and autophagy associated with p53/Bcl-2 modulation (Cerezo et al., 2013; Li et al., 2017a). Met is clinically applied only via oral administration. However, oral administration of Met is associated with gastrointestinal adverse effects such as indigestion and abdominal pains (Yu et al., 2015), which limits its clinical application.

Cubic phases are one of most popular transdermal drug delivery systems for decades. They own the complex spatial structure consisting of bicontinuous interpenetrating networks of water and amphiphiles (Yu et al., 2016). Thus, cubic phases can load a variety of polar or nonpolar regimens, including small molecular drugs, proteins, and vaccines (Rizwan et al., 2010). Moreover, the common matrix for cubic phases, such as glyceryl monooleate (GMO) and phytantriol (Nazaruk et al., 2014; Demurtas et al., 2015), intercalates with the extracellular liquid matrix of the skin and then disrupts the highly compacted structure (Hosmer et al., 2013; Simonetti et al., 2009). Therefore, cubic phases can enhance drugs transdermal permeation.

Reliable theoretical approximations and computational techniques are widely popular in pharmaceutical research with the development of fast computing technologies. Molecular docking was applied to explore the mechanism of physical stability on lacidipine amorphous solid dispersions (Sun et al., 2016). The molecular dynamics simulations were used to predict solubility parameters and miscibility of pharmaceutical compounds (Gupta et al., 2011). Molecular dynamics and dissipative particle dynamics simulations are integrated to investigate the loading/releasing of anti-cancer drug camptothecin in pH-sensitive amphiphilic copolymer (Luo & Jiang, 2012). Computational simulations for formulation optimization of cubic phases has not yet been reported.

In this study, Met-loaded cubic phases were explored and optimized by computational simulations and experiments. Here, the detailed in vitro/in vivo studies of Met-loaded cubic phases were performed. The high antimelanoma effect of the transdermal cubic phases was proved on the mouse melanoma models. Met-loaded cubic phases are a promising topically applied formulation for local treatment of skin cancers.

## Materials and methods

### Materials

Met was purchased from Jiangsu Hengrui Medicine Co., Ltd. (Lianyungang, China). GMO was supplied by Dansico Co., Ltd. (Boston, USA). All other materials were of pharmaceutical or analytical grade.

### Animals

Male Balb/c mice (20 ± 1 g, body weight) were supplied by the SPF Co., Ltd. (Beijing, China). All animal handling and surgical procedures were conducted in strict accordance with the NIH Guidelines for the Care and use of Laboratory Animals.

### *In silico* formulation optimization of met-loaded cubic phases

The cubic phase was transparent but stiff and viscous which limited its potential use as the delivery system (Peng et al., 2010). However, low viscous cubic phase could be obtained by adding organic solvents (propylene glycol, ethanol, polyethylene glycol, and N-methyl-2-pyrrolidone). Therefore, we selected the microstructure and the fluidity as two main indicators to screen the optimized formulation.

The basic components of Met-loaded cubic phases include Met, GMO, water, and ethanol. These molecules were put into the simulated system in our previous research (Yu et al., 2018). Briefly, the *in silico* formulation optimization procedure is described as follows. The whole simulation process was divided into two steps, fully atomistic molecular dynamics (MD) and dissipative particle dynamics (DPD) simulations. All simulations were performed in Materials Studio 7.0 (Accelrys Co., US).

Here, One GMO molecule was firstly divided into Fragments A and B that represented the hydrophilic glycerol and carboxyl group and the hydrophobic long carbon chain, respectively (Supplementary Figure 1). Whereafter, MD simulations were performed in order to obtain the Flory-Huggins parameters (χ) of binary components in all formulations (Luo & Jiang, 2012; Li et al., 2017b). DPD simulations were applied to build the microstructures of all Met/GMO/Ethanol/Water systems according to the Flory-Huggins parameters of binary components. The detailed operation process referred to our previous study (Yu et al., 2018). Finally, the optimized formulation can be screened based on the microstructure and water diffusivity of all liquid crystal systems.

### Preparation of met-loaded cubic phases

A Met solution was prepared by dissolving a certain amount of Met chloride in water at 50 °C. The Met solution was dropped into a GMO solution in ethanol at 50 °C under vortexing homogeneously with the ratio of water/GMO/ethanol provided by Table 1. The cubic phases obtained were sealed in a tube and ready for structure identification, *in vitro* permeation experiments and pharmacodynamics studies, etc.

**Table 1.** Composition and structures of liquid crystal systems.

Sample	GMO/ethanol/water/Met (w/w/w/w)	Spacing ratio	Structure	Lattice constant (Å)
F1	64/0/30/5	$\sqrt{6}\mathrm{:,}\sqrt{8}$	*Pn*3*m*	142
F2	64/2/30/5	$\sqrt{6}\mathrm{:,}\sqrt{8}$	*Pn*3*m*	136
F3	64/4/30/5	$\sqrt{6}\mathrm{:,}\sqrt{8}$	*Pn*3*m*	138
F4	64/6/30/5	$\sqrt{6}\mathrm{:,}\sqrt{8}$	*Pn*3*m*	134
F5	64/8/30/5	4:5	*Lamellar*	224
F6	64/10/30/5	3:4	*Lamellar*	198

### Characterization of the cubic phases

The structures of the samples were identified from their optical images and scattering characteristic at room temperature. Optical observations were conducted to acquire the images using a polarizing light microscopy (PLM, DMLP, Leica, Germany). The structures of the samples were determined in a small-angle X-ray scattering instrument (SAXS, SAXSee, Anton Paar, Austria) method referred to other reports (Evenbratt et al., 2013). Scattering intensities were plotted as a function of the reciprocal spacing.

### Permeation experiments

The dorsal skins of Balb/c mice were obtained after sacrifice. The vertical Franz diffusion cells with an effective diffusion area of 0.785 cm^2^ were used in the permeation study. The receptor compartment filled with saline of 10 ml was kept in a water bath maintained at 32 °C and stirred at 300 rpm. The model skin was assembled between donor cell and receptor cell with the stratum corneum (SC) side facing toward the donor cell. A Met solution or a Met-loaded cubic phase (sample F4) was put into the donor cell, which directly contacted the surface of SC. The administered dose was 1.5 g, containing 71.4 mg of Met and 85.5 mg of ethanol. At predetermined time points (0.5, 1, 2, 3, 4 and 5 h), 1 ml of saline was withdrawn from the receptor cell, and the same volume of fresh saline was injected into the receptor cell so as to reach the sink condition. The withdrawn samples were measured with the HPLC method illustrated as the reference (Ningrum et al., 2018). The cumulative Met permeation amount (Q_n_, μg/cm^2^) was calculated according to the Equation (1).

(1) $$Q_{n}=\frac{\left[C_{n}\cdot V+V_{0}\sum_{i=1}^{n-1}C_{i}\right]}{A}$$

where *V* (ml) is total volume of saline in the receptor cell, *C*_*n*_ (μg/ml) is the concentration of Sample *n*, *C*_*i*_ (μg/ml) is the concentration of Sample *i*, *V*_0_ (ml) is the volume of withdrawn saline and *A* (cm^2^) is the effective diffusion area. The *Q_n_* was plotted as a function of time.

The slop and intercept of the linear portion of the profile were presented as (Jss, μg·cm^−2^·h^−1^) and lag time (T_lag_, h), respectively. The activity of cubic phases was expressed as enhancement ratio (ER) that was the ratio of Q_5h_ in the cubic phase group to that in the solution group.

### Pharmacodynamic study

Every Balb/C mouse was subcutaneously injected into the hip using the B16 cell suspension (0.2 ml, 1 × 10^6^cells/ml). The xenograft volume (V) is measured with a caliper and calculated as 0.5 L × W^2^. where *L* is the largest superficial diameter and *W* is the smallest superficial diameter of the xenograft. The tumor sizes were measured and recorded every day. The formulations were initially applied to the mice at the tumor sites topically once daily after the tumor size reached about 50–100 mm^3^. The melanoma-bearing mice were divided to four groups of six mice in each group. Group I was composed of tumor-bearing mice without treatment and served as the control. The other groups included the tumor-bearing mice that were treated with the Met solutions (Group II), the blank cubic phase (Group III) and the Met cubic phase (Group IV). The mice were sacrificed after 10 days. The tumors were dissected and weighed to calculate the tumor inhibitory rates, which were defined as (*W*_blank_ − *W*_test_)/*W*_blank_×100%, where *W*_blank_ and *W*_test_ were the average tumor weights of the control group and the therapeutic groups, respectively.

### TUNEL immunohistochemical study

Some of the tumor tissues were preserved in all groups for tunel immunohistochemical study (Wang et al., 2018). First, tissue slides were treated with proteinase K (20 µg/ml) for 20 min at 25 °C. We equilibrated the tissue sections for 5 min using 100 µl of the kit’s equilibration buffer. FITC-12-d UTP Labeling Mix containing Recombinant Td T Enzyme was added to the sections, and the samples were incubated at 37 °C in a humidity chamber for 1 h and imaged using fluorescence microscopy.

### Statistical analysis

The statistical significance of the differences was analyzed by *t*-test or one-way analysis of variance (ANOVA) followed by Student–Newman–Keuls test. The value of *p* < .05 was considered significant.

## Results and discussion

### Flory–Huggins parameter

In the study, MD stimulations were carried out to estimate the Flory–Huggins parameter χ. The Flory–Huggins parameters (χ) between fragment B and other components were predicted by Supplementary Equation (1), because it is impossible for fragment B to form the hydrogen bonding with other molecules due to nonpolar structure. As listed in Supplementary Table 1, the estimated χ_B/H2O_=5.1 indicated fragment B was hydrophobic which made up the liquid lyophobic region of surfactant bilayer in the liquid crystal systems below. The estimated χ_B/ethanol_ = 0.5 suggested fragment B/ethanol was weakly miscible. In contrast, all other components can form mutually hydrogen bindings. Therefore, their values of χ were calculated by adopting Supplementary Equations (2) and (3), respectively. The χ_H2O/A_ was predicted to be −1.1, suggesting fragment A was insoluble in H_2_O but there was a certain amount of attraction force between them. With the number of molecules ratio of fragment A/ethanol decreased, the predicted Flory–Huggins parameters were more close to 0 (Supplementary Tables 2–6), indicating fragment A could be soluble in a certain amount of ethanol. The absolutes of χ_Met/ethanol_ decreased as the increase in ethanol content (Supplementary Tables 2–6), suggesting Met/ethanol could be easily miscible with the increasing of ethanol content.

### DPD modeling

**Figure 1.**
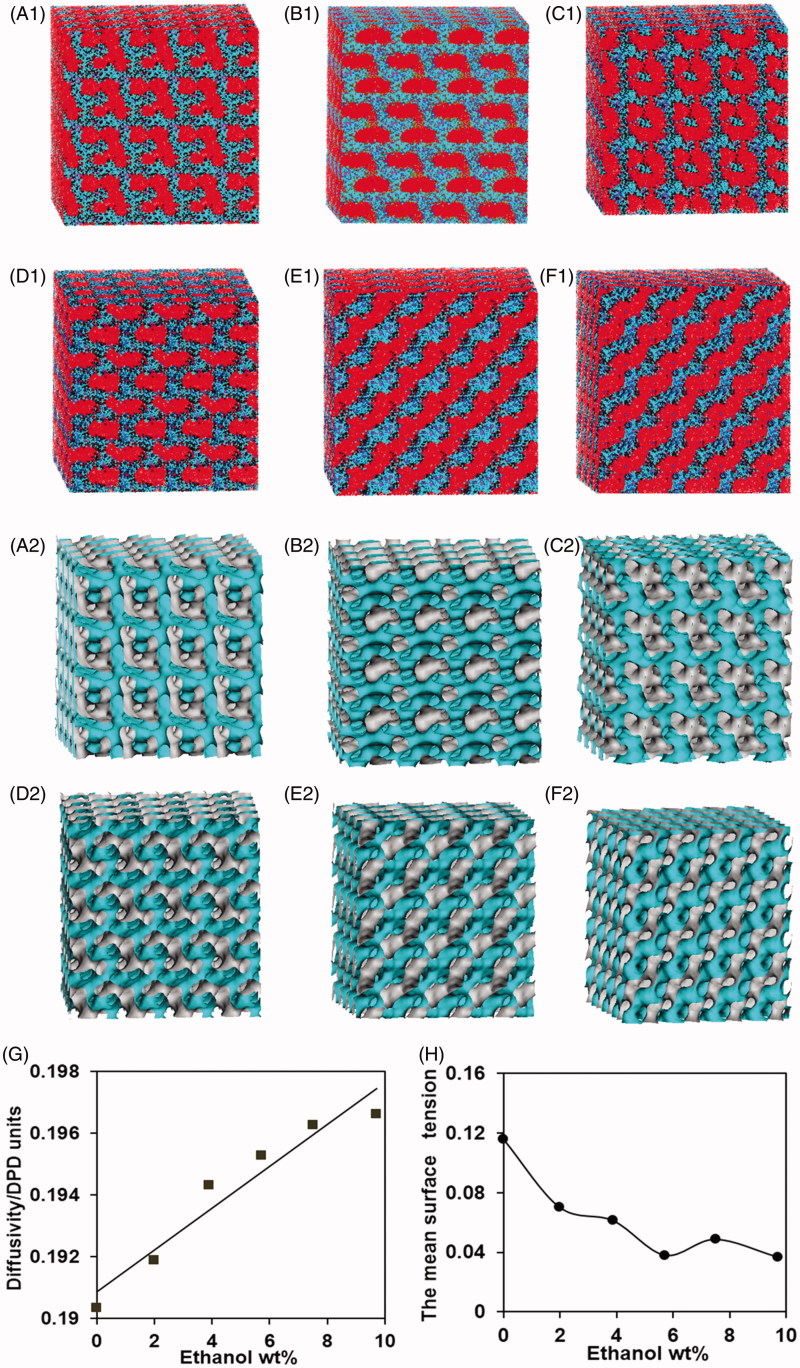
Characterization and representation of liquid crystal systems via DPD simulation. ‘dot-and-line’ models (series 1, A, B, Met, ethanol and water particle are presented in black, red, pink, blue and light blue, respectively.) and water particles’ isodensity surfaces (series 2, in light blue) at selected formulations: (A1, A2) sample F1, (B1, B2) sample F2, (C1, C2) sample F3, (D1, D2) sample F4, (E1, E2) sample F5, (F1, F2) sample F6. (G) Water diffusivity in liquid crystal systems as a function of ethanol concentration. (H) The mean surface tension in liquid crystal systems as a function of ethanol concentration.

Six samples, labeled as F1, F2, F3, F4, F5, and F6, were selected to build the internal structure of them using DPD stimulations. The calculated ‘dot-and-line’ models (series 1) and isodensity profiles of the water particles (series 2) for liquid crystal systems were shown in Figure 1. The GMO molecules in the systems arranged with hydrophobic groups (B particles) aggregated outside and the hydrophilic groups (A particles) oriented toward the water channels in which Met and ethanol molecules were distributed (series 1). The water particle isodensity surfaces usually represent the internal structure of liquid crystal systems (series 2). The samples F1-F4 exhibited extremely complex spatial organization and the bicontinuous interpenetrating networks of water channels. These results suggested they were cubic phases with the typical internal structures. The sample F5 appeared to have lamellar ordered array (series 1), but it was a non-lamellar phase with stalk intermediate structure (series 2). The result indicated the transition between liquid crystalline phases occurred at an ethanol/water wt ratio of 0.26. With observations from the perspective of surface and internal structure, the sample F6 had a highly ordered lamellar structure, suggesting the sample a lamellar phase.

The water diffusivity increased linearly with the increase of ethanol concentration (Figure 1(G)), suggesting adding ethanol could promote the fluidity of the liquid crystal systems. Moreover, the diffusivity of a water particle is a parameter representing the spatial structural complexity (Yang et al., 2006), and the internal structures of liquid crystal systems are more simple with the increasing of water diffusivity (Yang et al., 2006). Therefore, the results further demonstrated ethanol could simplify the internal structure of liquid crystal systems.

The surface tension between the liquid channels and the water channels was obtained from the various liquid crystal systems (Figure 1(H)). The surface tension could gradually reduce with the increasing amount of ethanol except at 7.7% (w/w) ethanol. This is because ethanol, being a polar solvent completely miscible with water, can be localized both at the interface thereby reducing the surface tension (Efrat et al., 2007), but a stalk intermediate formed usually requires relatively higher surface tension according to fusion mechanism (Lukatsky & Frenkel, 2004; Shillcock & Lipowsky, 2005).

In conclusion, the former four formulations were cubic phases, and sample F4 exhibited the proper viscosity among them. Thus, the sample F4 were finally selected as the optimized formulation.

### Characteristics of the cubic phase

**Figure 2.**
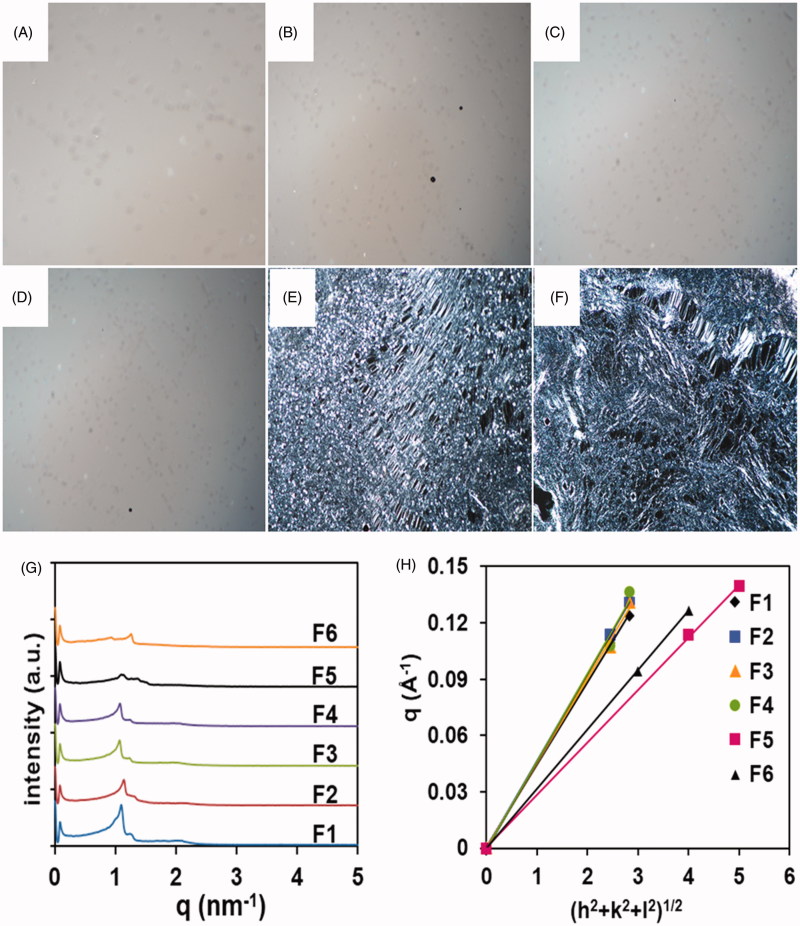
Characterization of liquid crystal systems by experimental measurements. PLM images of cubic phase (A, B, C, D) and lamellar phase (E, F) at room temperature (magnification ×400). SAXS diffraction patterns obtained from the six selected samples (G). Plots of the reciprocal d-spacings (q) as a function of the Miller indices, (h^2^ + k^2^ + l^2^)^1/2^, from the observed reflections in the SAXS diffraction patterns for the liquid crystal systems (H).

The images of F1, F2, F3, and F4 just displayed dark background under PLM (Figure 2(A–D)). All the values of the scattering vector q corresponding to the scattering peaks for four samples appear in the ratio of $\sqrt{6}：\sqrt{8}$ (Figure 2(G)), which seem to be consistent with Pn3m space group of cubic symmetry (Peng et al., 2010). Furthermore, the lattice parameters of the samples were calculated from the linear slope (Figure 2(H)). The lattice parameters of the four samples were calculated to be 142 Å, 136 Å, 138 Å, and 134 Å (Table 1), respectively. These results demonstrated the samples were cubic phases with the internal structure of Pn3m space group and various interlamellar distances. Moreover, the sample F5 had birefringent phenomenon with Maltese crosses by PLM images (Figure 2(E)). The SAXS measurement revealed a typical lamellar scattering pattern with two peaks following a 4:5 spacing relationship. The sample F5 was proved to be a lamellar phase. In the preparation process, the sample F5 could not be homogeneously mixed by the method of vortex. Thus, the difference appeared between the results of the experiment and the computational simulation. The sample F6 was slightly opaque and optically anisotropic with the presence of streak texture as illustrated by PLM images (Figure 2(F)), whose consistency resembled a weak gel with medium flow. SAXS scatterings (Figure 2(G)) showed two typical lamellar peaks with defined 3:4 spacing ratio. Therefore, the results confirmed sample F6 was also a lamellar phase.

The gels of F1, F2, F3, and F4 were homogeneous and transparent, where their flowing ability increased in turn. The F5 and F6 gels had better flowing ability than the F4 gel. However, the former two gels were not cubic phases, but lamellar gels. Therefore, the F4 gel was finally selected after considering the stable microstructure and proper fluidity. Furthermore, the computing technologies are extremely reliable for optimizing the formulations of the cubic phases.

### High efficient permeability of met by the cubic phase

**Figure 3.**
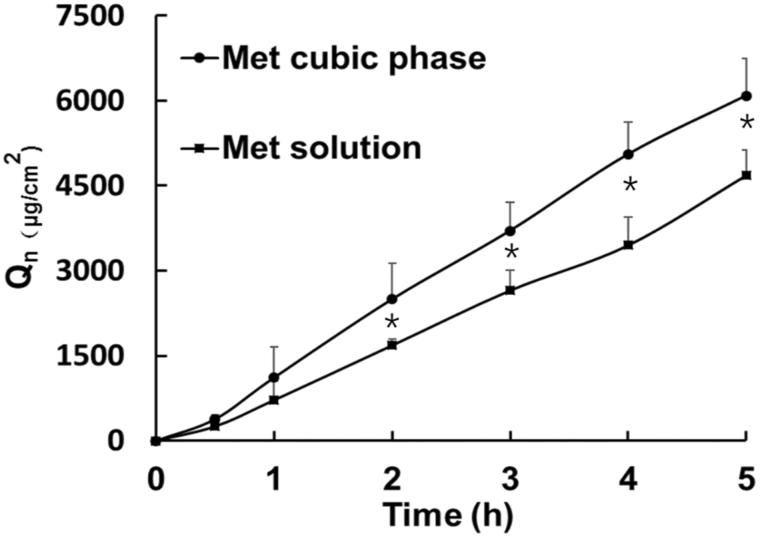
*In vitro* skin permeation profiles of Met from the cubic phases and the solutions versus time.

Skin permeation profiles present continuous increase trend as time prolonged, and Met skin permeation profile from the cubic phase was higher than that from the Met solution (Figure 3). As listed in Supplementary Table 7, there was significant difference on the cumulative amount per unit area within 5 hours between the cubic phase (*Q*_n_ = 6088 ± 663 μg/cm^2^) and the solution (*Q*_n_=4748 ± 517 μg/cm^2^, *p* < .05). In contrast to the Met solution (*J*_ss_=936.8 ± 121.5 μg**·**cm^−2^**·**h ^−1^), the steady-state flux of the cubic phase (*J*_ss_=1259.6 ± 312.6 μg**·**cm^−2^**·**h ^−1^) was obviously high. These results demonstrated the high Met transdermal improvement of cubic phases. Here, in our previous research, we explored the transdermal delivery mechanisms of cubic phases for Met with *in silico* and *in vitro* methods. Firstly, Met mainly diffuses from the water channels of cubic phases to the surface of stratum corneum. Next, GMO highly enhances Met permeation through skin by disturbing the interaction between Met and skin proteins, and disordering the arrangement of skin lipid molecules (Yu et al., 2018).

### Antimelanoma effects of the met-loaded cubic phases

**Figure 4.**
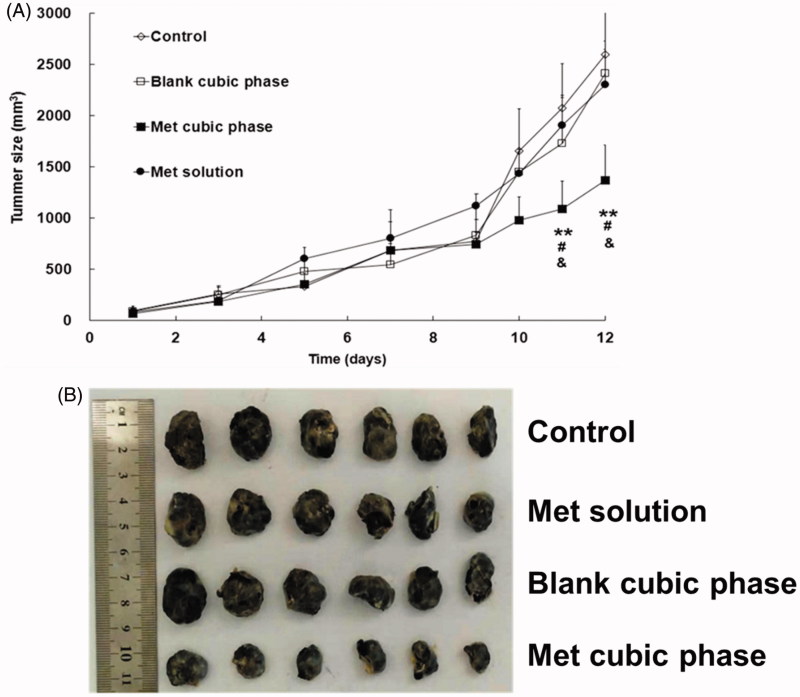
Profiles of the mouse tumor sizes following treatment with the Met formulations for melanoma therapy (A). Met cubic phase versus control, *p < .05, **p < .01. Met cubic phase versus Met solution, #*p* < .05. Met cubic phase versus blank cubic phase, &*p* < .05. Met cubic phase versus Blank cubic phase, &*p* < .05. The data are presented as the means ± SDs (*n* = 6). Images of the tumors following melanoma therapy with the Met formulations (B).

A significant difference started to appear on day 11, and maintained to the final day between the Met cubic phases (Group IV) and the control (Group I, Figure 4(A)). The result indicated the Met cubic phases had an obvious therapeutic effect on melanoma. In contrast, there was no significant difference between other two administration groups and control group, suggesting that both the Met solutions (Group II) and the blank cubic phases (Group III) had almost no anti-cancer effect. Obviously, the therapeutic results were strongly related to the Met permeation efficiencies of the formulations. In addition, the tumor weights of Groups I, II, III and IV were 1.73 ± 0.49, 1.54 ± 0.44, 1.63 ± 0.56 and 0.62 ± 0.18g, respectively, with the tumor inhibitory rates of 10.98%, 5.78% and 64.16%. Furthermore, the appearances of the tumors also exhibited significant differences between the groups (Figure 4(B)). These results further demonstrated the anti-cancer effect of the Met cubic phases was markedly high.

### Apoptosis and necrosis of melanoma cells

**Figure 5.**
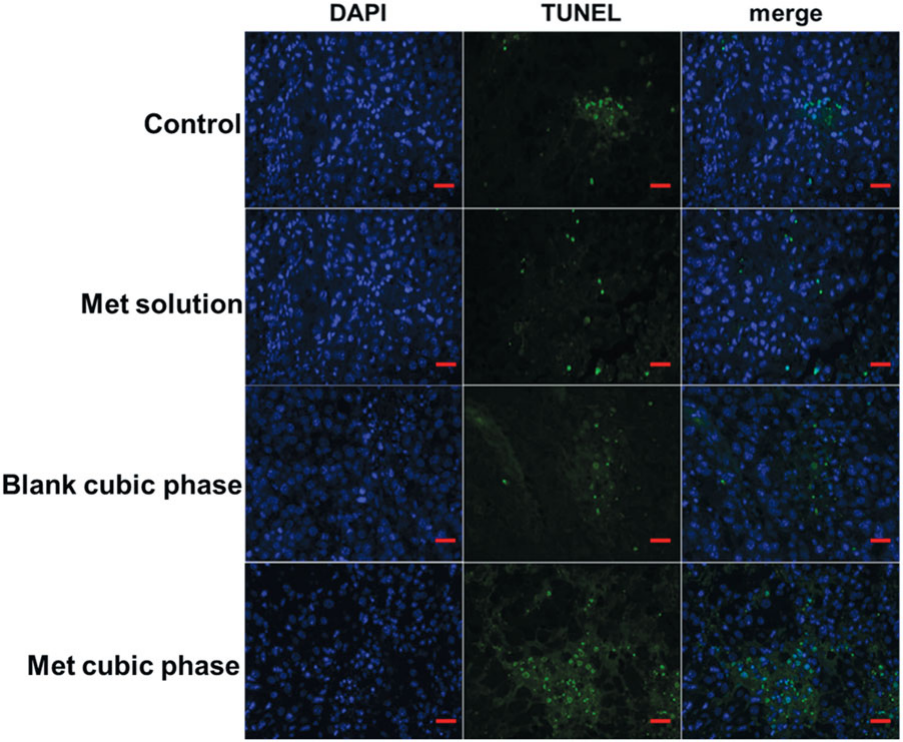
Representative micrographs of tumor tissue stained by TUNEL on day 12 post-administration. Scale bar, 100 μm.

To determine whether the pro-apoptotic effects of the Met cubic phases on melanoma cells occur *in vivo*, we performed tunnel assays on tumor sections from treated mice. A large positive tunnel staining area was observed in Group IV, while few areas were observed in other groups (Figure 5). These results indicated the Met cubic phases effectively promoted cell death. Met can inhibit the growth of melanoma with the induction of the cell cycle arrest and cell apoptosis (Janjetovic et al., 2011). Moreover, metformin-induced melanoma cell apoptosis is mainly associated with caspase activation, mitochondrial depolarization and oxidative stress (Janjetovic et al., 2011). In addition, the Met cubic phase has too high skin permeation (Yu et al., 2018). Therefore, the Met cubic phases can enhance apoptosis and necrosis of melanoma cells.

## Conclusions

Cubic phases are very popular transdermal delivery systems. Moreover, Met has recently been proved to have high therapeutic efficiency of antimelanoma and good skin permeation. Therefore, the novel contribution of this study was the optimization and the fabrication of a Met cubic phase for local therapy of melanoma. The GMO, the matrix for cubic phases, possess a rich array of temperature and composition sensitive behavior (Reese et al., 2015). It is extremely difficult for obtaining the optimized formulation of cubic phases quickly and accurately. Consequently, we developed a novel *in silico* modeling to optimize the Met formulation. The experimental methods demonstrated this technique was highly accurate. Most importantly, the Met cubic phases led to the reduction in the size of the melanoma due to the high skin permeation and cytotoxicity. Taken together, our work provides an alternative strategy in optimizing the formulations of drug delivery systems. Cubic phases are an ideal transdermal delivery system of Met as potential therapeutics for the clinical treatment of melanoma.

## Supplementary Material

Supplementary_Materials.docx
