# Supplementary material for: Transdermal metformin hydrochloride-loaded cubic phases: in silico formulation optimization, preparation, properties, and application for local treatment of melanoma
Source: Drug Deliv. 2019 Mar 24;26(1):376–83. doi: 10.1080/10717544.2019.1587046 (PMC6442100; doi:10.1080/10717544.2019.1587046)
Supplement: Supplementary_Materials.docx [file IDRD_A_1587046_SM9073.docx]

**Supplementary Materials**

**Transdermal metformin hydrochloride-loaded cubic phases: in silico formulation optimization, preparation, properties, and application for local treatment of melanoma**

**Xiang Yu^a,b^, Sheng Lu^a*^, Wei Zhou^b^，Hongmei^b^， Yiguang Jin^b*^，Junhui Fu^b^**

^a^*The Affiliated Hospital of Huzhou Teachers College, 158 Guangchanghou Road, Huzhou 313000, China*

^b^*Department of Pharmaceutical Sciences, Beijing Institute of Radiation Medicine, 27 Taiping Road, Beijing 100850, China*

*Corresponding authors. Yiguang Jin, e-mail: jinyg@sina.com; Sheng Lu, e-mail: hulsph@sina.cn.

1. *Molecular dynamics simulation*


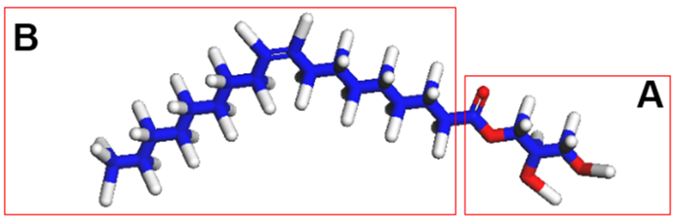


**Figure 1.** Chemical structure of GMO divided into Fragments A and B.

The Flory-Huggins parameter between the components *i* and *j* in nonpolar systems can be calculated with equation (1) if no special interactions such as hydrogen bonding occurs.

(1)

where *δ_i_* and *δ_j_* are solubility parameters of *i* and *j*, respectively, equal to the square root of cohesive energy densities; *V*_ref_ is reference volume; *R* is gas constant; and *T* is temperature.

The Flory-Huggins parameter *χ*_ij_ of polar components or the components with special interactions can be approximately estimated from equations (2) and (3).

(2)

(3)

where *∆E_mix_* is the energy of mixing *i* and *j*; *Φ*_i_ and *Φ*_j_ are the volume fraction of *i* and *j*; , and are the cohesive energy densities of *i*, *j* and their mixture, respectively.

(ρ = 5，T = 328 K) (4)

where a_ij_ is a repulsive parameter between i and j beads. In the context, equation (4) bridges the gap between atomistic MD and DPD simulations.

For each system, the simulation box size was 20×20×20 rc with a periodic boundary condition. The total beads were 27302 and the spring constant C was chosen as 4.0. To obtain the steady results, 50000 DPD steps had been adopted with a time step of 1.0 ps.

**Table 1：**Molecular parameters of Sample F1 examined by MD simulations at 328K.

| GMO/ethanol/water/Met (w/w/w/w) | Components | Number of molecules | χ | a |
| --- | --- | --- | --- | --- |
|  | H_2_O/ Met | 664/12 | 0.19 | 16.8 |
|  | H_2_O/ A | 664/72 | -1.1 | 15.0 |
| 64/0/30/5 | H_2_O/ B | 664/72 | 5.10 | 23.9 |
|  | Met/A | 48/288 | -1.22 | 14.7 |
|  | Met/ B | 48/288 | 4.87 | 23.5 |
|  | A/ B | 72/72 | 1.07 | 18.0 |

**Table 2**：Molecular parameters of Sample F2 examined by MD simulations at 328K.

| GMO/ethanol/water/Met (w/w/w/w) | Components | Number of molecules | χ | a |
| --- | --- | --- | --- | --- |
|  | H_2_O/ Met | 664/12 | 0.19 | 16.8 |
|  | H_2_O/ A | 664/72 | -1.1 | 15.0 |
|  | H_2_O/ B | 664/72 | 5.10 | 23.9 |
|  | H_2_O/ ethanol | 664/17 | -0.07 | 16.4 |
| 64/2/30/5 | Met/A | 48/288 | -1.22 | 14.7 |
|  | Met/ B | 48/288 | 4.87 | 23.5 |
|  | A/ B | 72/72 | 1.07 | 18.0 |
|  | A/ ethanol | 72/17 | 0.77 | 17.6 |
|  | B/ ethanol | 72/17 | 0.48 | 17.2 |
|  | Met/ ethanol | 48/68 | -1.51 | 14.3 |

**Table 3**：Molecular parameters of Sample F3 examined by MD simulations at 328K.

| GMO/ethanol/water/Met (w/w/w/w) | Components | Number of molecules | χ | a |
| --- | --- | --- | --- | --- |
|  | H_2_O/ Met | 664/12 | 0.19 | 16.8 |
|  | H_2_O/ A | 664/72 | -1.1 | 15.0 |
|  | H_2_O/ B | 664/72 | 5.10 | 23.9 |
|  | H_2_O/ ethanol | 664/34 | -0.07 | 16.4 |
| 64/4/30/5 | Met/A | 48/288 | -1.22 | 14.7 |
|  | Met/ B | 48/288 | 4.87 | 23.5 |
|  | A/ B | 72/72 | 1.07 | 18.0 |
|  | A/ ethanol | 72/34 | 0.61 | 17.4 |
|  | B/ ethanol | 72/34 | 0.48 | 17.2 |
|  | Met/ ethanol | 48/136 | -1.39 | 14.5 |

**Table 4**：Molecular parameters of Sample F4 examined by MD simulations at 328K.

| GMO/ethanol/water/Met (w/w/w/w) | Components | Number of molecules | χ | a |
| --- | --- | --- | --- | --- |
|  | H_2_O/ Met | 664/12 | 0.19 | 16.8 |
|  | H_2_O/ A | 664/72 | -1.1 | 15.0 |
|  | H_2_O/ B | 664/72 | 5.10 | 23.9 |
|  | H_2_O/ ethanol | 664/51 | -0.07 | 16.4 |
| 64/6/30/5 | Met/A | 48/288 | -1.22 | 14.7 |
|  | Met/ B | 48/288 | 4.87 | 23.5 |
|  | A/ B | 72/72 | 1.07 | 18.0 |
|  | A/ ethanol | 72/51 | 0.34 | 17.0 |
|  | B/ ethanol | 72/51 | 0.48 | 17.2 |
|  | Met/ ethanol | 48/204 | -0.96 | 15.1 |

**Table 5**：Molecular parameters of Sample F5 examined by MD simulations at 328K.

| GMO/ethanol/water/Met (w/w/w/w) | Components | Number of molecules | χ | a |
| --- | --- | --- | --- | --- |
|  | H_2_O/ Met | 664/12 | 0.19 | 16.8 |
|  | H_2_O/ A | 664/72 | -1.1 | 15.0 |
|  | H_2_O/ B | 664/72 | 5.10 | 23.9 |
|  | H_2_O/ ethanol | 664/68 | -0.07 | 16.4 |
| 64/8/30/5 | Met/A | 48/288 | -1.22 | 14.7 |
|  | Met/ B | 48/288 | 4.87 | 23.5 |
|  | A/ B | 72/72 | 1.07 | 18.0 |
|  | A/ ethanol | 72/68 | 0.22 | 16.8 |
|  | B/ ethanol | 72/68 | 0.48 | 17.2 |
|  | Met/ ethanol | 48/272 | -0.64 | 15.6 |

**Table 6**：Molecular parameters of Sample F6 examined by MD simulations at 328K.

| GMO/ethanol/water/Met (w/w/w/w) | Components | Number of molecules | χ | a |
| --- | --- | --- | --- | --- |
|  | H_2_O/ Met | 664/12 | 0.19 | 16.8 |
|  | H_2_O/ A | 664/72 | -1.1 | 15.0 |
|  | H_2_O/ B | 664/72 | 5.10 | 23.9 |
|  | H_2_O/ ethanol | 664/85 | -0.07 | 16.4 |
| 64/10/30/5 | Met/A | 48/288 | -1.22 | 14.7 |
|  | Met/ B | 48/288 | 4.87 | 23.5 |
|  | A/ B | 72/72 | 1.07 | 18.0 |
|  | A/ ethanol | 72/85 | -0.04 | 16.7 |
|  | B/ ethanol | 72/85 | 0.48 | 17.2 |
|  | Met/ ethanol | 48/340 | -0.28 | 16.1 |

**Table 7.** The permeation parameters of Met from two formulations

| Samples | Q_5h_  (μg/cm^2^) | J_ss_  (μg/cm^2^/h) | ER | T_lag_  (h) |
| --- | --- | --- | --- | --- |
| Cubic phase | 6088±663^*^ | 1259.6±312.6^*^ | 1.34 | 0.08 |
| Solution | 4748±517 | 936.8±121.5 | 1 | 0.17 |

Each value was presented as mean ± SD (n = 3). * p < 0.05.
